# Triptolide Suppresses Tongue Squamous Cell Carcinoma Progression via PI3K–AKT Pathway Inhibition: An Integrative Network Pharmacology and Experimental Validation Study

**DOI:** 10.1002/kjm2.70283

**Published:** 2026-08-26

**Authors:** Shan‐Shan Wang, Hong‐Cheng Wei, Lin Jiang, Yi‐Sen Shao

**Affiliations:** ^1^ Department of Oral and Maxillofacial Surgery, Key Laboratory of Oral Diseases of Traditional Chinese Medicine Jiangxi University of Traditional Chinese Medicine Nanchang China; ^2^ School of Stomatology Nanchang University Nanchang China

**Keywords:** network pharmacology, PI3K–AKT signaling pathway, tongue squamous cell carcinoma (TSCC), triptolide

## Abstract

Tongue squamous cell carcinoma, the most aggressive subtype of oral squamous cell carcinoma, which is associated with high mortality, frequent recurrence, and early lymph‐node metastasis. Triptolide, a bioactive diterpenoid from *Tripterygium wilfordii*, exhibits broad anticancer activity. Although its inhibitory action on OSCC has been established, its specific molecular regulatory mechanisms in TSCC, a distinct and aggressive subtype, remain largely underexplored. The present study investigates the regulatory role of triptolide in the PI3K–AKT signaling pathway, which is a critical axis intimately linked to tumor survival and metastasis. A network‐pharmacology workflow was used to intersect triptolide putative targets with TSCC‐related genes. Core hubs were identified with Cytoscape; KEGG and GO analyses highlighted the PI3K–AKT axis as the top enriched pathway. Binding kinetics were quantified by molecular docking and surface plasmon resonance (SPR). Cellular assays (CCK‐8, Transwell, wound‐healing, Western blot and immunofluorescence) validated the predictions. We retrieved 389 common targets and shortlisted eight key nodes (JUN, MAPK1, MAPK3, AKT1, TP53, BCL2, STAT3, MYC). SPR revealed high‐affinity binding of triptolide to AKT1 and PI3K. Functionally, triptolide dose‐dependently suppressed TSCC cell proliferation, invasion, and migration, downregulated PI3K–AKT signaling proteins, and reduced the colocalization and phosphorylation levels of p‐PI3K and p‐AKT. This study is the first to demonstrate the pivotal role of the PI3K–AKT signaling pathway in mediating the antitumor effects of triptolide in tongue squamous cell carcinoma. Our findings thereby provide a novel theoretical foundation for the development of traditional Chinese medicine‐based therapeutic strategies targeting this malignancy.

## 1 Introduction

Tongue squamous cell carcinoma (TSCC), a distinct subset of squamous cell carcinoma, remains the most frequently encountered malignant tumor in oral and maxillofacial surgery [1]. Its etiology is well characterized and predominantly reflects personal habits and environmental exposures, most notably betel‐quid chewing, tobacco smoking, alcohol consumption, and the malignant transformation of chronic traumatic ulcers subjected to prolonged exogenous irritation [2]. TSCC carries a comparatively low overall survival rate, exhibits a high incidence of local recurrence, and demonstrates a striking propensity for early cervical lymph‐node metastasis, often manifesting when the primary lesion is still clinically small [3]. Contemporary advances in high‐resolution imaging and refined pathological sampling techniques have undoubtedly improved the early detection rate of the disease, and contemporary surgical methodologies—incorporating microvascular free‐tissue transfer and meticulous neck dissection—have achieved a high degree of technical maturity; nevertheless, the ultimate cure rate for TSCC has not improved markedly in recent years [4]. The current standard multimodal treatment approach is accompanied by considerable postoperative morbidity, including substantial tongue‐tissue defects, impaired articulation, dysphagia, reduced gustatory function, and compromised airway protection. In addition, the systemic side effects of conventional cytotoxic agents—myelosuppression, nephrotoxicity, neurotoxicity, and mucositis—together with the emergence of drug‐resistant tumor clones, continue to erode patients' quality of life, relegating it to a level that remains far from satisfactory [5, 6, 7]. Consequently, the active exploration of novel pharmacological agents that combine potent antitumor efficacy with minimal adverse effects and low susceptibility to resistance is of paramount clinical importance.

The development of traditional Chinese medicine (TCM) is currently advancing at a rapid pace, demonstrating significant advantages in synergistic enhancement, toxicity reduction, and functional rehabilitation [8, 9]. These benefits are fully realized in the treatment research of numerous diseases, particularly, oncological conditions [10, 11]. Central to this strategy is triptolide, a diterpenoid lactone purified from the medicinal vine *Tripterygium wilfordii*. The compound possesses a broad pharmacophore that confers potent anti‐inflammatory, immunomodulatory, and anti‐neoplastic activities [12]. Mechanistically, triptolide suppresses tumor‐cell proliferation, triggers mitochondrial and death‐receptor‐mediated apoptosis, imposes cell‐cycle checkpoints, and disrupts angiogenic crosstalk. These pleiotropic effects are underpinned by its ability to modulate endogenous reactive oxygen species (ROS) and nitric oxide (NO) pools, to interfere with RNA‐polymerase‐II‐mediated transcription, and to rewire survival pathways governed by Mcl‐1, the Bcl‐2/Bax rheostat, NF‐κB, and NFAT [13, 14, 15, 16].

Relevant reports indicate that triptolide exhibits significant inhibitory effects on pancreatic cancer [17], colorectal cancer [18], and lung cancer [19], yet research on their application in treating squamous cell carcinoma of the tongue remains scarce. Although studies have demonstrated the anti‐tumor activity of triptolide in oral squamous cell carcinoma (OSCC), its potential molecular mechanisms continue to be explored. Collectively, these studies demonstrate the complexity of triptolide's actions and their potent antitumor efficacy, suggesting a multipathway regulatory mechanism underlies their antitumor effects.

This study represents the first instance of network pharmacology being employed to deduce the gene targets involved in the interaction between triptolide and TSCC. Utilizing KEGG and GO enrichment analyses, the PI3K–AKT pathway was identified as a potential mechanism of action, subsequently validated through experimental investigation. As it remains unclear whether and how triptolide inhibits TSCC by modulating the PI3K–AKT pathway—which plays a central role in tumor survival and metastasis [20, 21]—this study aims to identify novel targets for triptolide in TSCC and investigate its effects on TSCC cell proliferation, invasion, and epithelial‐mesenchymal transition (EMT) through regulation of this pathway. This will further refine the molecular regulatory network underpinning TCM treatment for TSCC.

## 2 Materials and Methods

### 2.1 Data Acquisition

Triplolide‐related targets were harvested from two publicly accessible repositories: the Traditional Chinese Medicine Systems Pharmacology Database and Analysis Platform (TCMSP, https://www.tcmsp‐e.com/) and the Comparative Toxicogenomics Database (CTD, https://ctdbase.org/). Using the keyword “triptolide,” 853 nonredundant human genes were retrieved.

To define the TSCC target landscape, the identical keyword string “tongue squamous cell carcinoma” was interrogated across four complementary sources: GeneCards (https://www.genecards.org/), OMIM (https://www.omim.org/), the Therapeutic Target Database (https://db.idrblab.net/ttd/) and DisGeNET (https://disgenet.com/). All identifiers were then mapped to official gene symbols and UniProt accessions ( *Homo sapiens* ) to ensure uniformity prior to downstream analyses.

### 2.2 Construction of the Protein–Protein Interaction (PPI) Network and Selection of Core Targets

In R Studio, invalid and redundant entries were removed from the pooled triptolide and TSCC target lists. The cleaned genes were plotted with JVENN (https://jvenn.toulouse.inra.fr/app/example.html) to visualize the overlap [22]. The shared targets were imported into STRING to generate a PPI network, after which the network was loaded into Cytoscape 3.10.2. Using the CytoNCA plug‐in, topological scoring was performed to extract the core hub genes that link the drug to the disease.

### 2.3 GO and KEGG Enrichment Analyses

The clusterProfiler R package was used to execute GO enrichment (biological process, BP; cellular component, CC; molecular function, MF) on the core targets. KEGG pathway analysis was then run to identify the signaling cascades most significantly associated with the hub genes.

### 2.4 Molecular Docking Analyses

The triptolide MOL2 structure was retrieved from PubChem. Candidate protein 3‐D structures were downloaded from the RCSB Protein Data Bank (http://www.rcsb.org) and pre‐processed (water deletion, hydrogen addition, charge calculation) in PyMOL 2.4 and AutoDock Tools 1.5.6. AutoDock Vina 1.1.2 performed the docking, and the resulting poses were rendered with PyMOL 2.6.

### 2.5 SPR Binding Kinetics Analysis

To determine the binding kinetics between triptolide and its target protein, SPR experiments were performed using the Biacore Insight Evaluation 5.0.18.22102 system. Recombinant human AKT1 (UniProt: P31749, designed and synthesized by IPODIX, Wuhan, China) and PI3K proteins (UniProt: P42336, designed and synthesized by IPODIX, Wuhan, China) were immobilized onto channels of a CM5 sensor chip via the standard amine coupling method. The analyte triptolide was serially diluted in running buffer to yield a concentration gradient. The experiments were performed at 25°C with a flow rate of 30 μL/min. Following reference subtraction, the sensorgrams were fitted using a 1:1 Langmuir binding model to determine the association rate constant (*k*_a_), dissociation rate constant (*k*_d_), and equilibrium dissociation constant (KD = *k*_d_/*k*_a_). Data are given as mean ± standard error of the mean, *n* = 3 independent replicates.

### 2.6 Cell Culture

Human oral keratinocyte (HOK) cells (Gineo Biotechnology Co. Ltd., Guangzhou, China) and the TSCC lines SCC‐9 and CAL 27 (Wuhan Boyuan Biotechnology Co. Ltd., Wuhan, China) were maintained in Dulbecco's modified Eagle medium (DMEM) containing 10% fetal bovine serum (FBS) and 1% penicillin–streptomycin. Cultures were incubated at 37°C in 5% CO_2_; logarithmic‐phase cells were used for all assays.

### 2.7 Cytotoxicity Test

Triptolide (T107400, Aladdin, Shanghai, China), the PI3K agonist 740Y‐P (GC16157, GLPBIO, USA) and the PI3K inhibitor LY294002 (20 μM; HY‐10108, MedChemExpress, USA) were dissolved in dimethyl sulfoxide (DMSO) to generate concentrated stock solutions that were diluted in complete medium immediately before use; the final DMSO concentration in all working solutions was ≤ 0.1% (v/v). An equal volume of 0.1% DMSO was added to the negative‐control (NC) group in every experiment. CAL 27, SCC‐9, and HOK cells were seeded at 1 × 10^4^ cells per well in 96‐well plates, exposed to 0–320 nM triptolide and incubated until approximately 85% confluence. After two PBS washes, cell viability was quantified with the CCK‐8 assay (450 nm absorbance). Each concentration was tested in triplicate in three independent experiments. To assess whether PI3K activation rescues triptolide‐induced cytotoxicity, CAL 27 and SCC‐9 cells were treated with IC_50_ concentrations of triptolide alone or in combination with 740Y‐P (18 μM) for 24 h. Cell viability was then determined by CCK‐8 assay as described above.

### 2.8 Cell Invasion Assay

Transwell inserts (8 μm pores) were coated with Matrigel. Serum‐free medium containing 5 × 10^4^ SCC‐9 or CAL 27 cells was placed in the upper chamber, while the lower chamber received medium supplemented with 10% FBS. After 24 h incubation, inserts were rinsed with PBS, fixed in 4% paraformaldehyde for 20 min, and stained with 0.1% crystal violet for 30 min. Matrigel on the upper surface was gently removed, and invading cells were photographed at 200× magnification. Cell counts were quantified with ImageJ; each experiment was performed in triplicate and repeated three times.

### 2.9 Colony Formation Assay

Logarithmic‐phase cells were seeded at 2 × 10^3^ cells per well in six‐well plates and cultured for 14 day under the indicated triptolide concentrations. Colonies were rinsed twice with PBS, fixed with 4% paraformaldehyde for 30 min, stained with 0.1% crystal violet for 15 min, air‐dried, and photographed. Clusters ≥ 50 cells were scored as colonies; numbers were quantified with ImageJ. Each experiment was performed in triplicate for every cell line.

### 2.10 Cell Scratch Assay

Sterile 200‐μL pipette tips were used to create straight scratches across confluent monolayers (≈90%) in six‐well plates pre‐marked on the underside; equal pressure was applied to ensure uniform gap width. Floating cells were removed by gentle PBS washes, and the 0 h image was captured at ×200 magnification. Fresh medium containing the designated triptolide doses was then added. After 24 h incubation, the same fields were reimaged. Relative wound closure was calculated with ImageJ as [(area₀ *h* − area_2_₄ *h*)/area₀ *h*] × 100%. Each experiment was repeated three times.

### 2.11 Western Blot (WB)

CAL 27 and SCC‐9 cells (4 × 10^5^/well) were grown to 80%–90% confluence in six‐well plates, exposed to the indicated triptolide concentrations for 24 h, washed with ice‐cold PBS, and lysed in RIPA buffer containing protease and phosphatase inhibitors. Lysates were cleared by centrifugation (12,000*g*, 15 min, 4°C) and protein concentrations were determined with the BCA assay. Samples (30 μg) were mixed with 4× Laemmli buffer, heated (100°C, 5 min), resolved on 10% SDS‐PAGE, and electro‐transferred to PVDF membranes. After blocking with 5% BSA in TBST (1 h, room temperature), membranes were incubated overnight at 4°C with primary antibodies against the proteins of interest; GAPDH served as loading control. Membranes were washed three times with TBST (10 min each), incubated with HRP‐conjugated secondary antibodies (room temperature, 2 h), washed again, and developed with ECL reagent. Bands were imaged on a chemiluminescence system and quantified by ImageJ. Each experiment was performed in triplicate. All primary antibodies against PI3K, phospho‐PI3K, AKT, and phospho‐AKT were purchased from HuaAn Biotechnology Co. Ltd. (Hangzhou, China). Antibodies against GAPDH, E‐Cadherin and N‐Cadherin were obtained from Abmart (Shanghai, China). All antibodies were used according to the manufacturers' instructions.

### 2.12 Immunofluorescence Colocalization

Sterile coverslips were placed in a 24‐well plate before seeding. CAL 27 or SCC‐9 cells were plated at 1 × 10^4^ cells per well and grown to 80% confluence. After brief washing with PBS, cells were fixed with 4% paraformaldehyde (room temperature, 10 min), permeabilised with 0.1% Triton X‐100 (10 min), and blocked with 5% bovine serum albumin (30 min). Coverslips were incubated overnight at 4°C with primary antibodies against phosphorylated PI3K (p‐PI3K) and phosphorylated AKT (p‐AKT), washed three times with PBS, then exposed to Alexa‐Fluor‐conjugated secondary antibodies for 1 h at room temperature in the dark. Following three additional PBS washes, nuclei were counter‐stained with DAPI for 10 min. Coverslips were mounted on glass slides and imaged with a fluorescence microscope. Positivity was quantified with ImageJ. Data are presented as mean ± SEM from three independent experiments.

### 2.13 Statistical Analysis

All data in this research were examined using GraphPad Prism v9.5 and are presented as the mean ± standard deviation. Variations between different groups were assessed utilizing one‐way analysis of variance (with the Tukey's method employed for multiple comparison analysis) or Student's *t*‐test. Differences were considered statistically significant when *p* < 0.05 (*p < 0.05, **p < 0.01, ***p < 0.001, ****p < 0.001).

## 3 Results

### 3.1 Identification of Cross‐Targets Between Drugs and Diseases, and Screening of PPI Networks and Core Targets

**FIGURE 1:**
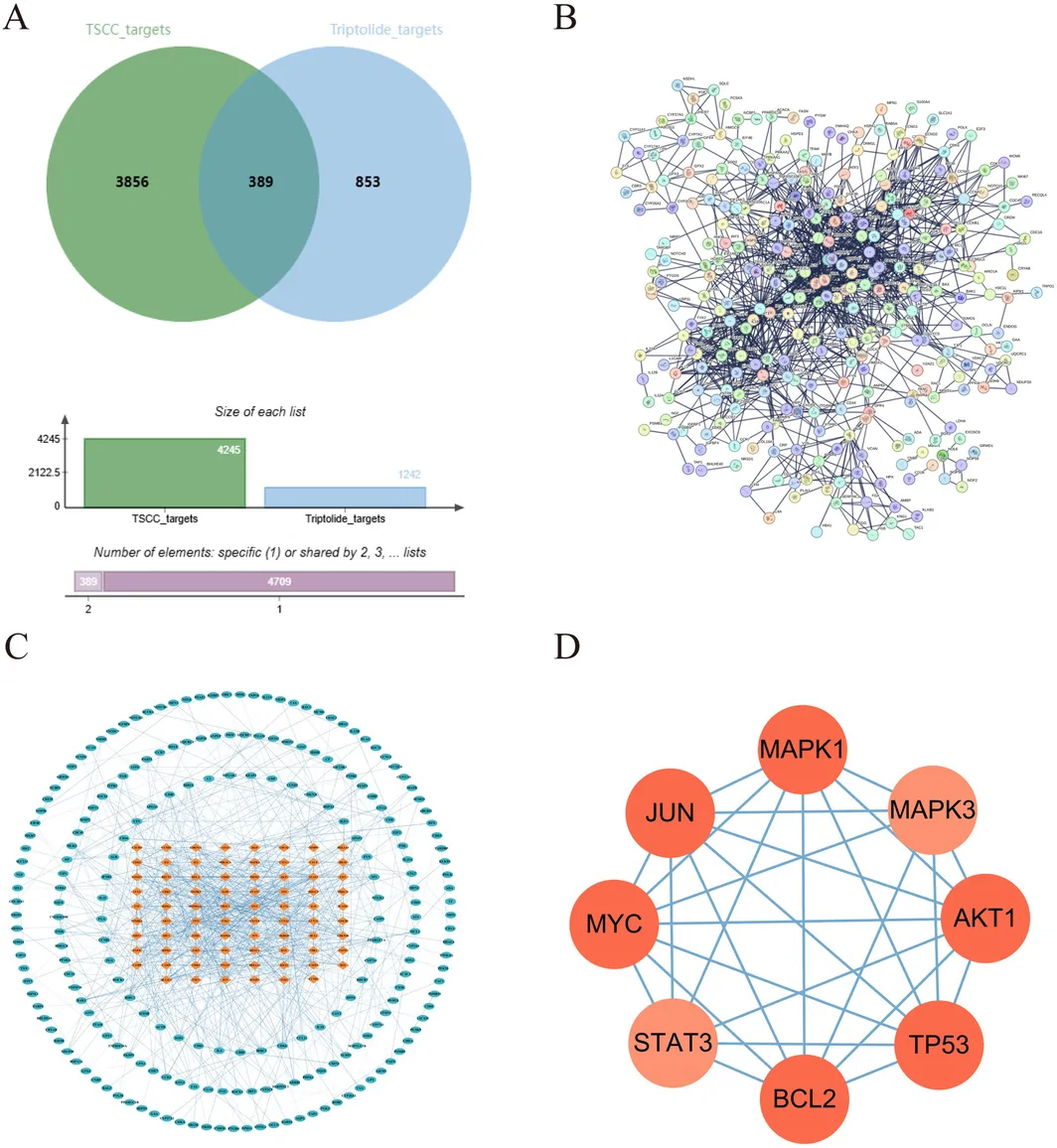
PPI network and core targets: (A) A Venn diagram depicting 389 cross‐targets between drugs and diseases. (B) The PPI map of the 389 cross‐targets. (C) Visualization of the 389 cross‐targets presented as blue circular rectangles. (D) Eight core targets screened by topological methods.

Through systematic mining of four public databases (GeneCards, OMIM, TTD, and DisGeNET), a total of 3856 TSCC‐related targets were retrieved, while 853 triptolide‐related targets were harvested from TCMSP and CTD. These two pools were overlapped in JVENN, which returned 389 cross‐targets common to both the drug and the disease (Figure 1A). To visualize the interactome landscape, the 389 cross‐targets were uploaded to the STRING platform with the species restricted to *H. sapiens* . The resultant PPI network contained 389 nodes and 7842 edges, giving an average node degree of 40.3 (Figure 1B). The complete interaction file was exported in TSV format and imported into Cytoscape 3.10.2. Using the CytoNCA plug‐in, we calculated five topological parameters—degree, betweenness, closeness, eigenvector and local average connectivity—to quantify the centrality of each node. Targets whose degree value exceeded the median were retained in the first round; the same filter was applied in a second iterative screening to distil the most influential hubs. After these two rounds of selection, eight high‐degree core targets were ultimately prioritized: JUN, MAPK1, MAPK3, AKT1, TP53, BCL2, STAT3, and MYC (Figure 1C,D). These proteins constitute the central scaffold through which triptolide is predicted to exert its therapeutic effects on TSCC.

### 3.2 KEGG and GO Enrichment Analyses

**FIGURE 2:**
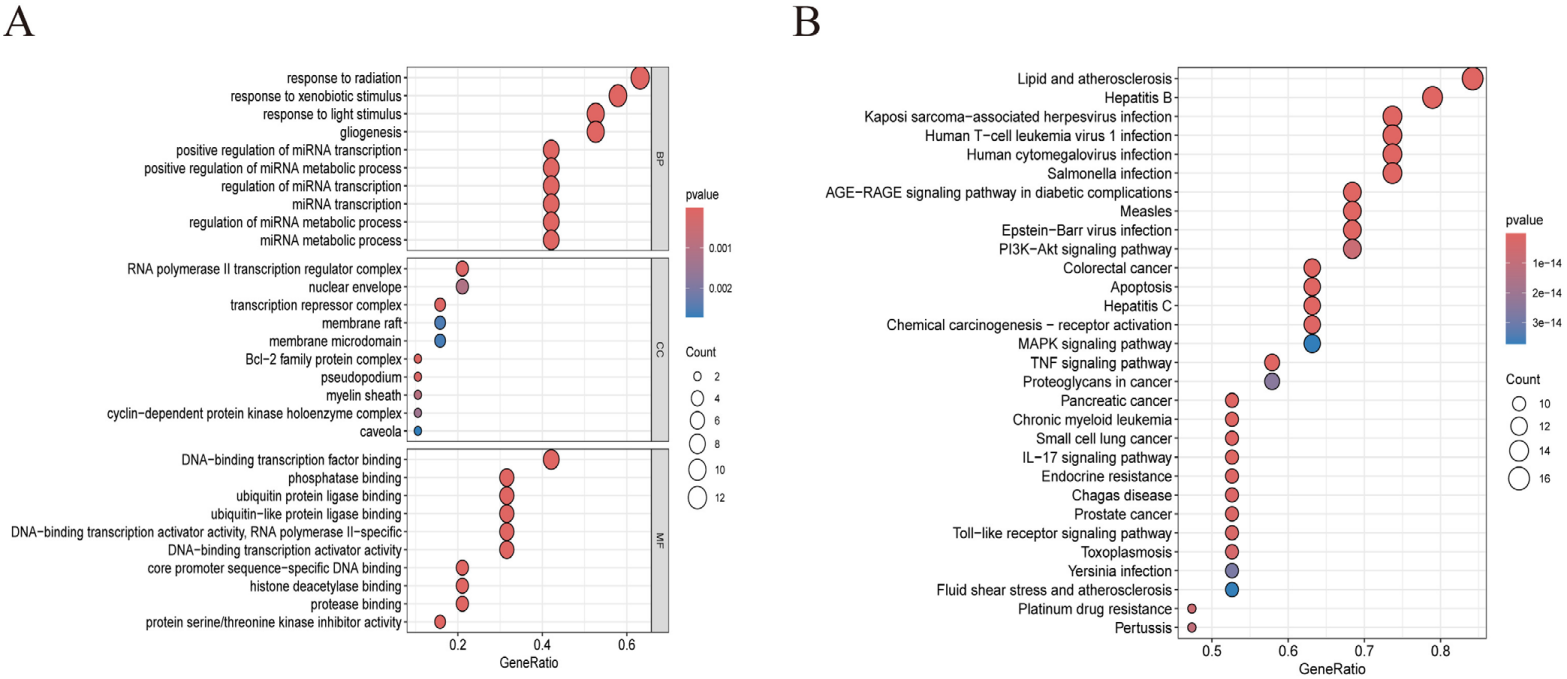
(A) GO enrichment analysis; (B) KEGG pathway analysis.

To decode the BPs and signaling cascades through which triptolide acts on TSCC, the eight core targets were subjected to GO and KEGG enrichment analyses with the clusterProfiler R package. Among the top 30 GO terms (Figure 2A), the most prominent BP included response to radiation, response to xenobiotic stimulus, and gliogenesis; CC terms were enriched in RNA Polymerase II transcription regulator complex, nuclear envelope, and membrane raft; MF annotations highlighted DNA‐binding transcription factor binding, phosphatase binding, and protein serine/threonine kinase inhibitor activity.

The KEGG pathway bubble plot (Figure 2B) revealed 30 significantly enriched pathways (adjusted *p* < 0.01). Among these, the PI3K–AKT signaling pathway was ranked 10th by GeneRatio. Although several higher‐ranked pathways were primarily associated with infectious diseases (e.g., Pertussis, Yersinia infection, Toxoplasmosis), the PI3K–AKT pathway is one of the most central and well‐documented regulators of tumor cell proliferation, migration, and invasion.

Numerous independent studies have validated that hyperactivation of PI3K–AKT is an early and persistent driver of TSCC progression; consequently, we hypothesized that this axis constitutes the principal conduit through which triptolide restrains the malignant phenotype of TSCC cells. Subsequent experiments were therefore designed to verify this bioinformatic prediction.

### 3.3 Molecular Docking Analysis

**FIGURE 3:**
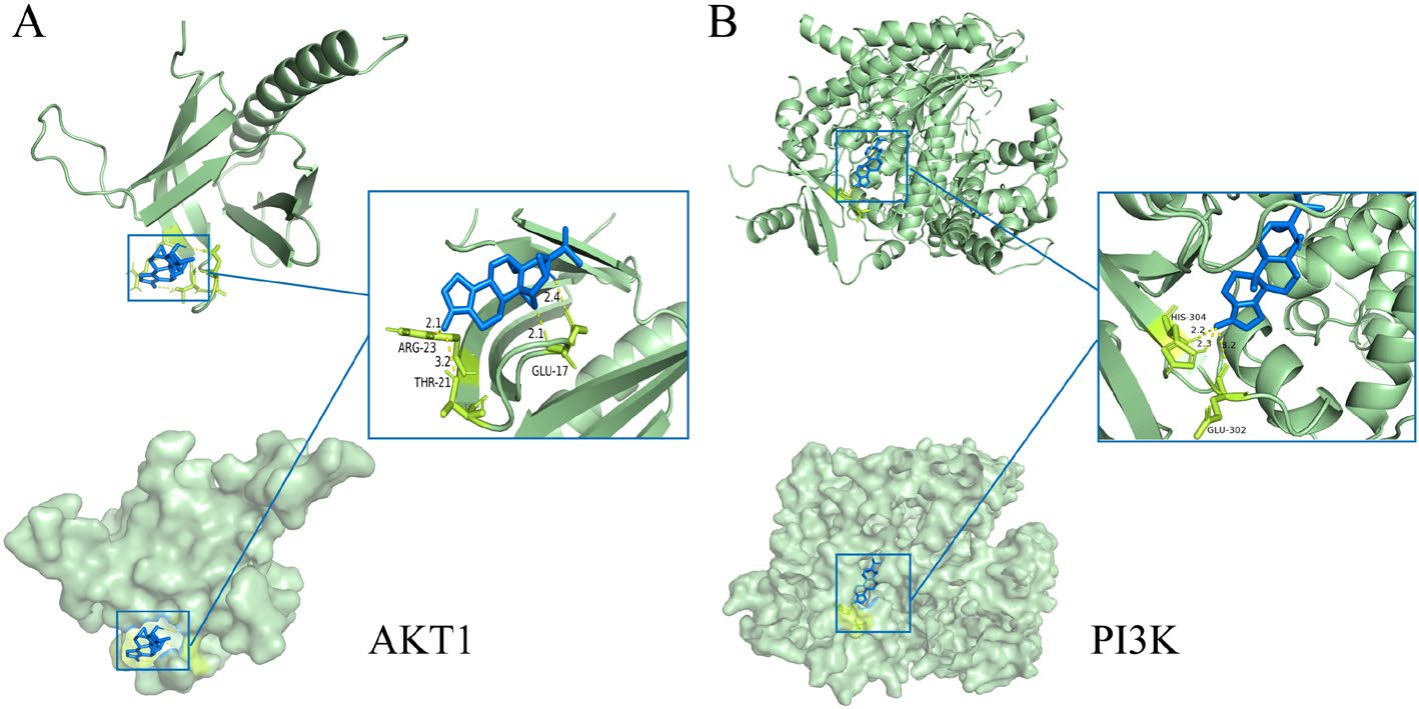
(A) Molecular docking was performed using triptolide as the receptor and AKT1 as the ligand; (B) molecular docking was carried out with triptolide serving as the receptor and PI3K acting as the ligand.

To estimate the binding propensity of triptolide toward the hub proteins of the predicted PI3K–AKT axis, we performed in silico docking against AKT1 (PDB: 1UNP) and PI3K (PDB: 7JIU). The lowest‐energy poses are displayed in Figure 3. AKT1 established a hydrogen‐bond network with the diterpenoid lactone ring of triptolide (Figure 3A), yielding a binding free energy (Δ*G*) of −7.7 kcal mol^−1^. PI3K displayed an even more favorable interaction profile (Δ*G* = −8.6 kcal mol^−1^), in which the ligand is deeply embedded within the ATP‐binding pocket and forms multiple hydrophobic contacts as well as a key hydrogen bond with the hinge region (Figure 3B). Because Δ*G* values more negative than −5 kcal mol^−1^ are generally accepted to indicate spontaneous and stable complex formation, both docking results suggest that triptolide may bind within the ATP‐binding pockets of AKT1 and PI3K, providing a structural basis for further experimental validation.

### 3.4 To Verify the Binding Affinities of Triptolide for AKT1 and PI3K

**FIGURE 4:**
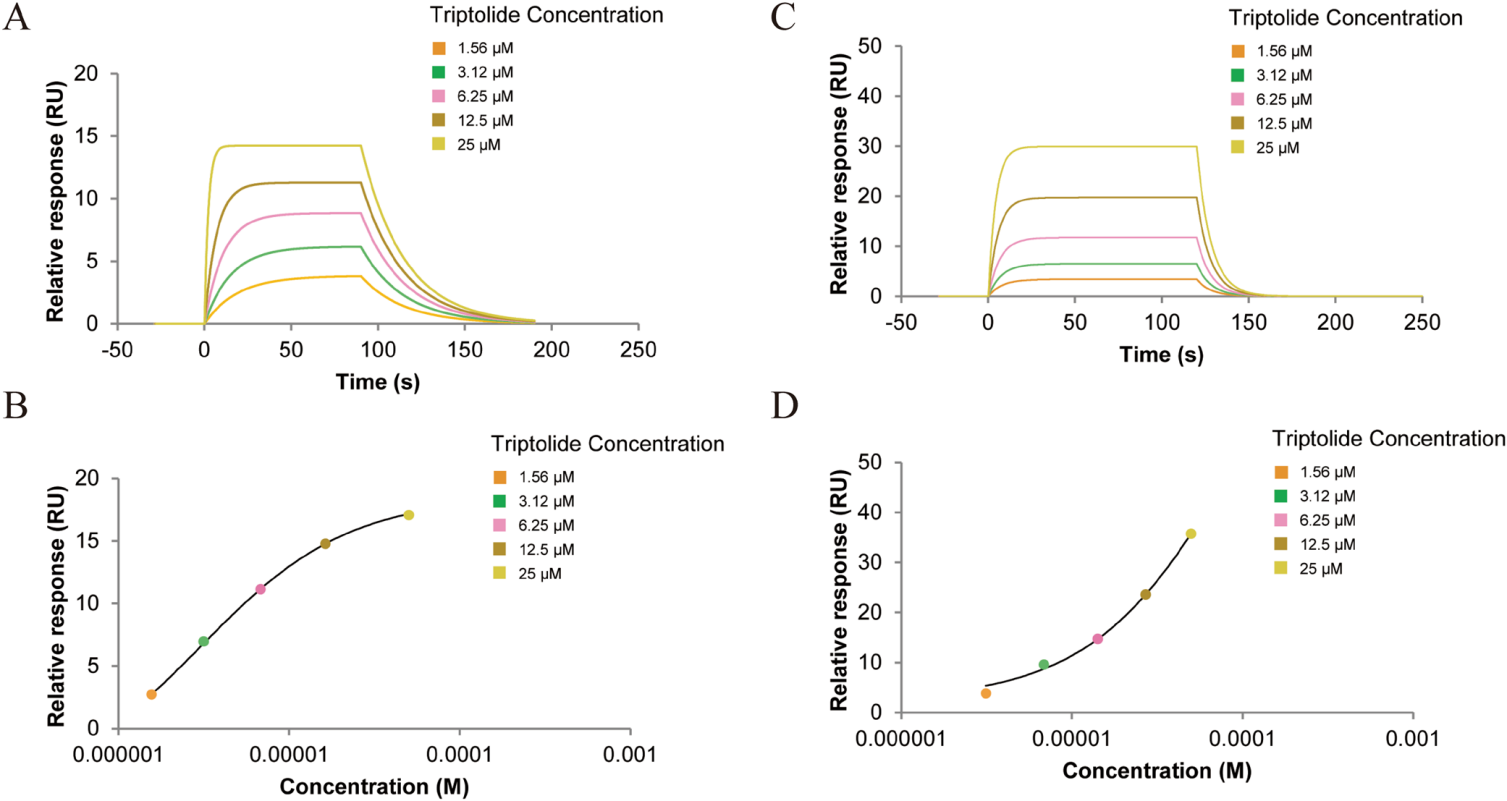
The result of SPR analysis for triptolide molecular and proteins encoded by AKT1 (A, B) and PI3K (C, D). Data are given as mean ± standard error of the mean, *n* = 3 independent replicates.

SPR assays were further employed to dissect the binding kinetics and molecular affinities between triptolide and AKT1 as well as PI3K. Fitted experimental data revealed that the equilibrium dissociation constant (KD) for the drug‐AKT1 protein interaction was 3.00 × 10^−6^ M and the drug‐PI3K protein interaction was 1.05 × 10^−4^ M. The features of the kinetic curves indicated the formation of a stable complex between the two (Figure 4) and detailed experimental results are provided in Table S1.

### 3.5 The Inhibitory Effect of Triptolide on the Phenotype of TSCC Cells

**FIGURE 5:**
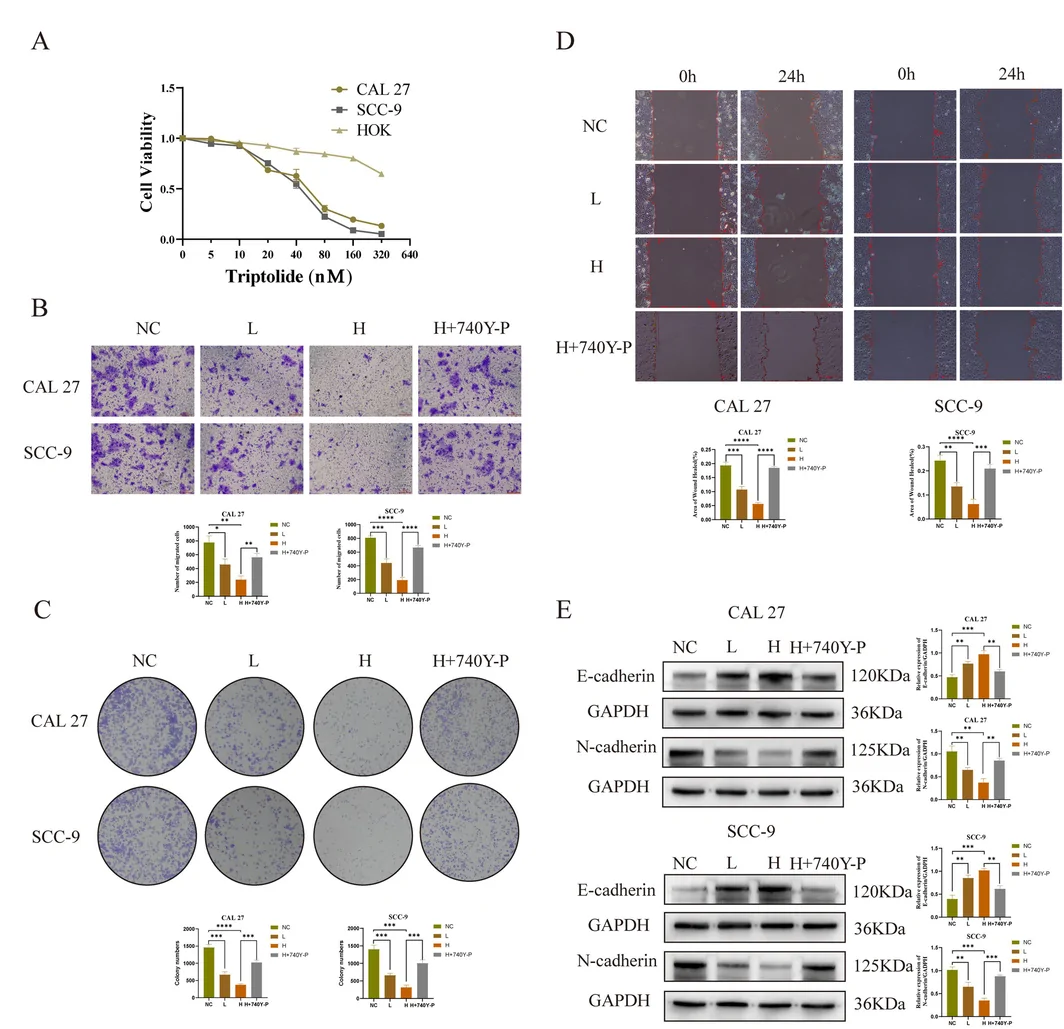
Effects of triptolide on the phenotypes of TSCC cells: (A) The CCK‐8 assay was used to evaluate the biological safety of triptolide and select the effective concentration. (B) The Transwell invasion assay was conducted to validate the impact of triptolide on the cell invasion ability. (C) The colony formation assay was performed to confirm the effect of triptolide on the cell proliferation ability. (D) The scratch assay was carried out to verify the influence of triptolide on the cell migration ability. (E) The expression levels of EMT‐related molecules following treatment with triptolide were characterized using WB analysis. Data are given as mean ± standard error of the mean, *n* = 3 independent replicates. *p < 0.05, **p < 0.01, ***p < 0.001, ****p < 0.0001.

First, we employed the CCK‐8 assay to assess the cytotoxicity of triptolide. To this end, we cultured TSCC cell lines (CAL 27 and SCC‐9) and human normal oral keratinocytes (HOK). The cell viability of these cells was measured following treatment with different concentrations of triptolide (0, 5, 10, 20, 40, 80, 160, 320 nM), and their respective half‐maximal inhibitory concentration (IC_50_) values were determined. The results indicated that as the concentration increased, the survival rates of all three cell types declined, and a marked difference was observed between TSCC cells and HOK (Figure 5A). Upon data analysis, the IC_50_ values of CAL 27 and SCC‐9 were determined to be 36.98 and 42.85 nM, respectively, whereas that of HOK was 448.3 nM. This finding demonstrates that triptolide exhibits selective cytotoxicity within a specific concentration range. Consequently, in subsequent experiments, we selected 0, 1/2 IC_50_, and IC_50_ concentrations of triptolide for the NC, L, and H groups, respectively, to carry out the experimental investigations. To validate the specific inhibitory effect of the drug on the PI3K–AKT signaling pathway, an additional experimental group treated with the PI3K pathway‐specific agonist 740Y‐P (GC16157, GLPBIO) + H was incorporated. The working concentration of 740Y‐P was set at 18 μM, which was adopted based on previously validated concentrations that effectively activate the PI3K–AKT pathway in the same cell model and are commonly used in rescue experiments [23].

The findings of the Transwell invasion assay revealed that, in comparison to the NC group, the cell invasive capacity decreased markedly as the drug concentration increased (Figure 5B). The colony formation assay demonstrated that the cell proliferation rate was significantly reduced as the drug concentration increased (Figure 5C). In the wound healing assay, it was observed that the cell migration rate at 24 h decreased significantly with an increasing drug concentration (Figure 5D). Furthermore, to establish the role of triptolide in the EMT process of TSCC, this study assessed the expression levels of EMT‐associated molecules. The results (Figure 5E) indicated that triptolide was capable of downregulating the mesenchymal protein N‐cadherin while upregulating E‐cadherin, and this effect exhibited concentration‐dependence. Collectively, the above experiments demonstrated that triptolide significantly suppressed the proliferation, invasion, and migration capacities of TSCC cells. In contrast, the combination treatment of triptolide and 740Y‐P significantly reversed these inhibitory effects on TSCC cells (*p* < 0.05). 740Y‐P significantly rescued the triptolide‐reduced cell viability (Figure S4D, *p* < 0.01). To further confirm that the observed anti‐migratory and anti‐invasive effects were not merely due to general cytotoxicity, we repeated the Transwell invasion, colony formation, and wound healing assays at a sublethal concentration of 1/4 IC_50_. At this concentration, cell viability remained above 85% based on the CCK‐8 results (Figure 5A). Triptolide significantly inhibited cell invasion (Figure S1), colony formation (Figure S2), and wound healing (Figure S3) at 1/4 IC_50_. These results support that triptolide exerts specific inhibitory effects on TSCC cell migration and invasion beyond general cytotoxicity. As shown in Figure S4A–C, treatment with the PI3K inhibitor LY294002 suppressed cell invasion, colony formation, and migration in both cell lines, recapitulating the inhibitory effects of triptolide.

### 3.6 Triptolide Downregulated the Expression of Proteins Associated With the PI3K–AKT Signaling Pathway

**FIGURE 6:**
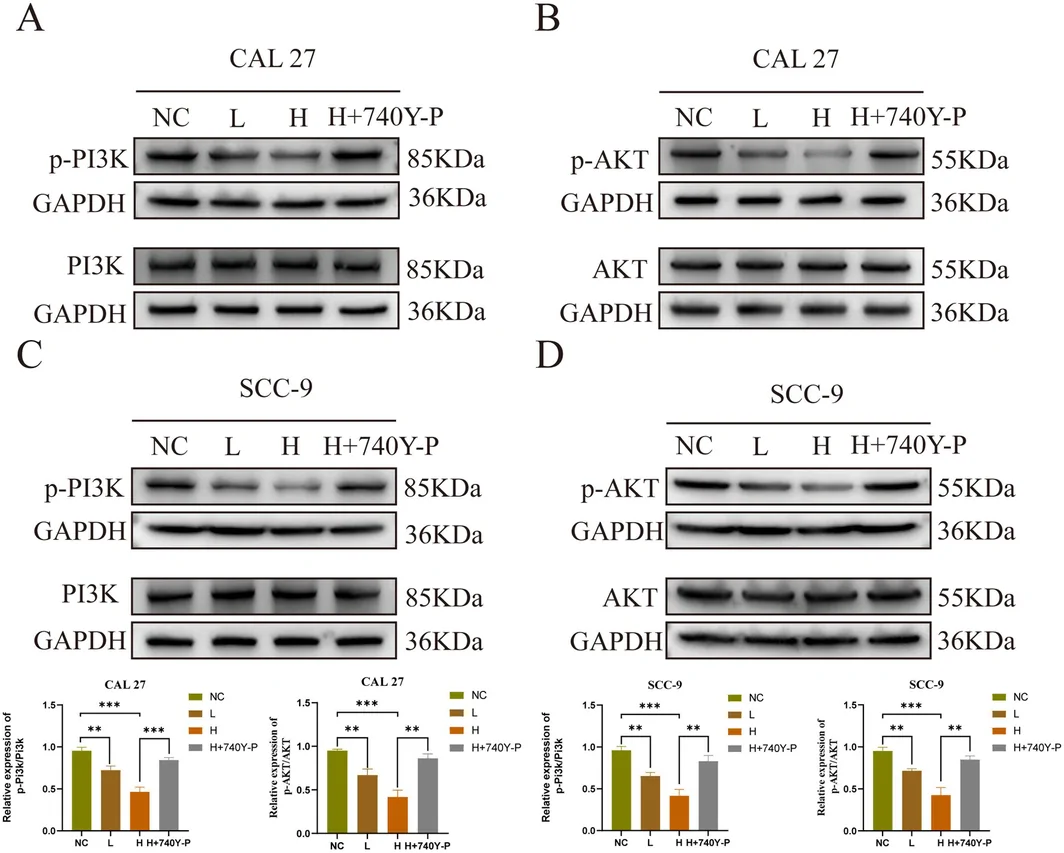
Triptolide suppresses the expression of proteins associated with the PI3K–AKT pathway: (A, B) Expression of proteins associated with the PI3K–AKT pathway in CAL 27 cells upon treatment with varying concentrations of triptolide. (C, D) Expression of proteins associated with the PI3K–AKT pathway in SCC‐9 cells upon treatment with varying concentrations of triptolide. Data are given as mean ± standard error of the mean, *n* = 3 independent replicates. *p < 0.05, **p < 0.01, ***p < 0.001, ****p < 0.0001.

Based on the KEGG and GO enrichment analyses of relevant core targets, we identified the PI3K–AKT signaling pathway as a potential mechanism through which triptolide exerts its effects on TSCC. To investigate this further, we utilized WB assays to examine the expression of proteins associated with this pathway. As presented in Figure 6, an increase in the concentration of triptolide led to a decrease in the expression levels of p‐PI3K and p‐AKT. These results suggest that triptolide inhibits the expression of proteins involved in the PI3K–AKT signaling pathway. Concurrently, the results from the H + 740Y‐P group validated the efficacy of the agonist at the molecular level. We assessed the phosphorylation levels of key proteins in the PI3K–AKT signaling pathway. Relative to the control group, triptolide treatment significantly decreased the levels of p‐PI3K and p‐AKT. In contrast, cotreatment with 740Y‐P led to a significant recovery of these phosphorylated protein signals.

### 3.7 Triptolide Suppresses the Activity of the PI3K–AKT Pathway and Reduces Colcalization Between Upstream and Downstream Molecules

**FIGURE 7:**
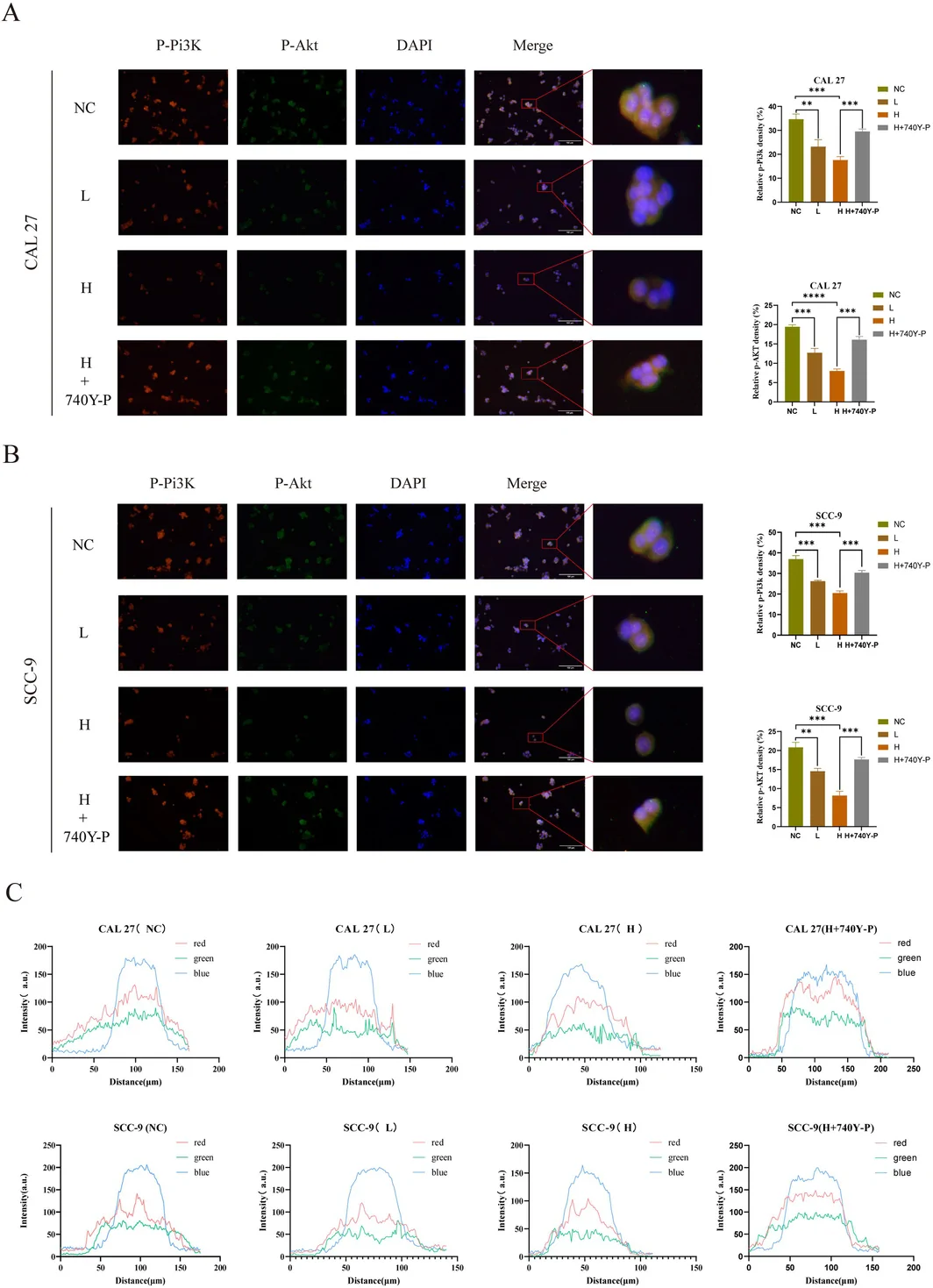
Colocalization of p‐PI3K and p‐AKT in TSCC cells: (A) Staining of p‐PI3K (red), p‐AKT (green), and the nucleus (blue) in CAL 27 cells. The merged image demonstrates the colocalization of p‐PI3K (red) and p‐AKT (green) within the cytoplasm. (B) Staining of p‐PI3K (red), p‐AKT (green), and the nucleus (blue) in SCC‐9 cells. The merged image reveals the colocalization of p‐PI3K (red) and p‐AKT (green) in the cytoplasm. (C) A colocalization line graph generated from the analysis of the merged images of p‐PI3K and p‐AKT in CAL 27 and SCC‐9 cells. Data are presented as mean ± standard error of the mean, *n* = 3 independent replicates. *p < 0.05, **p < 0.01, ***p < 0.001, ****p < 0.0001. Colocalization indicates spatial overlap and does not imply direct protein–protein interaction.

We employed immunofluorescence assays to assess the impact of triptolide on the activity of the PI3K–AKT signaling pathway. As depicted in the fluorescence images presented in Figure 7, Cy3‐labeled p‐PI3K elicited the distribution of red fluorescence within the cells, while FITC‐labeled p‐AKT induced the distribution of green fluorescence inside the cells.

In the cells of the negative control (NC) group, which were not treated with the drug, distinct red and green fluorescence signals were observable. These two signals exhibited extensive colocalization within the cytoplasm (the area where yellow fluorescence was superimposed), suggesting that the PI3K–AKT signaling pathway was highly active under basal conditions and that p‐PI3K and p‐AKT were spatially closely associated. As the concentration of the drug increased, the expression levels (fluorescence intensity) of both p‐PI3K and p‐AKT decreased significantly (Figure 7A,B). More critically, the colocalization analysis (performed using the Plot Profile function in ImageJ) revealed that the degree of colocalization between p‐PI3K and p‐AKT also diminished with increasing drug concentration (Figure 7C). However, cotreatment with 740Y‐P significantly reversed this inhibitory effect.

These findings indicate that triptolide not only inhibits the phosphorylation‐activated states of PI3K and AKT but also reduces the intracellular colocalization of p‐PI3K and p‐AKT, consistent with decreased pathway activity.

## 4 Discussion

TSCC is the most frequently encountered malignant neoplasm of the oral and maxillofacial region and is distinguished by an unusually high mortality and recurrence burden. Despite advances in imaging, surgical technique, and adjuvant radio‐chemotherapy, the 5‐year cure rate remains below 50% [24]. Radical resection—still the cornerstone of therapy—often sacrifices considerable volumes of normal tongue tissue, leading to permanent articulation, mastication, and swallowing dysfunction together with visible cosmetic deformity. Recurrent disease poses an even greater dilemma: repeat surgery is technically demanding, carries higher peri‐operative morbidity, and rarely translates into meaningful prolongation of overall survival. Exploring pharmacotherapy for TSCC may represent a breakthrough in tackling this complex challenge.

Multiple, nonoverlapping mechanisms have been ascribed to triptolide. It can transcriptionally silence c‐Myc and mitochondrial hexokinase‐II, thereby activating the BAD/BAX–caspase‐3 cascade and provoking gasdermin‐E‐mediated pyroptosis in head‐and‐neck cancer cells [25]. Independent work shows that triptolide binds and inactivates peroxiredoxin‐2, elevating intracellular ROS and driving endoplasmic‐reticulum‐stress‐dependent autophagy [26]. In cervical carcinoma models the compound triggers ferroptotic death through the NRF2/GPX4/xCT axis [27], whereas in osteosarcoma, it suppresses the DNA‐damage response and sensitizes cells to genotoxic chemotherapy [28, 29]. Very recent evidence further identifies the HNF1A/SHH pathway as a triptolide target capable of reversing paclitaxel resistance [30]. Collectively, these studies portray triptolide as a pleiotropic agent that can both eradicate cancer cells and dismantle chemo‐resistance networks, making it an attractive candidate for TSCC‐directed or combination therapy.

Although the clinical utilization of triptolide is constrained by issues such as poor solubility, low bioavailability, and substantial toxicity to the liver, kidneys, and gastrointestinal tract, with the advancement of materials science, these challenges may be gradually addressed. Minnelide, a water–soluble prodrug of triptolide, has been evidenced to exhibit potent anti–tumor activity across a wide range of cancer models [31]. In a breast cancer study, nanoparticles coated with hyaluronic acid (HA) and loaded with triptolide were employed. The results demonstrated no signs of toxicity in a mouse model of transplanted tumors [32]. In research focusing on liver cancer, it was further validated that nanoliposomes encapsulated with triptolide not only exert augmented anti‐hepatic tumor effects but also markedly mitigate the liver‐associated toxicity and systemic toxicity related to triptolide [33].

Our data now demonstrate that triptolide suppresses TSCC proliferation, colony formation, invasion, and migration in a concentration‐dependent manner. This finding is consistent with previous reports in OSCC, further confirming its anti‐tumor potential. However, distinct from these studies, our work is the first to identify the PI3K–AKT signaling pathway as a central mechanism of triptolide action in TSCC. While Hao et al. [34] predicted multiple triptolide targets through network pharmacology, their core focus centered on the JAK–STAT and MAPK pathways. Kuo et al. [35] emphasized triptolide's regulation of PD‐L1 within the immune microenvironment. Chen et al. [36], meanwhile, approached the subject from the perspective of non‐coding RNAs, elucidating the role of the ceRNA regulatory network. It is worth noting that the PI3K–AKT pathway may cross‐talk with other signaling cascades, such as JAK–STAT and MAPK pathways [37], which are known to be involved in tumor resistance and compensatory activation. Although the present study did not experimentally investigate these interactions, future studies using combinatorial inhibitors or phosphoproteomics could explore whether PI3K–AKT inhibition by triptolide leads to compensatory activation of JAK–STAT or MAPK pathways in TSCC.

First, we specifically focused on TSCC, a more aggressive subtype, and confirmed the specific involvement of the PI3K–AKT pathway. By integrating network pharmacology, GO/KEGG enrichment and molecular docking, we identified AKT1 as a high‐affinity triptolide target and the PI3K–AKT axis as the most probable signaling conduit. Ligand‐receptor complex formation was subsequently validated by SPR, confirming that the diterpenoid occupies the ATP‐binding pockets of both PI3K and AKT1. Once engaged, the cascade is interrupted at its apex: generation of PIP3 is blunted, AKT phosphorylation is suppressed and downstream effectors such as mTOR, GSK‐3β, BAD, and FOXO remain in their inactive state. Because the PI3K–AKT network intersects with HIF, MAPK, Notch and Wnt pathways, its silencing simultaneously dampens angiogenic, proliferative and antiapoptotic transcriptional programs that are essential for TSCC survival [38]. Equally important, hyperactivation of this pathway has been repeatedly linked to cisplatin resistance through increased DNA‐damage repair, decreased oxidative stress and enhanced drug efflux [39, 40]; consequently, its pharmacological restraint by triptolide may resensitize refractory tumors to platinum‐based regimens. Second, we innovatively introduced SPR technology to validate the direct binding affinity between triptolide and PI3K, AKT1 proteins. Furthermore, we have confirmed that triptolide can downregulate the expression of proteins associated with the PI3K–AKT signaling pathway through WB analysis combined with immunofluorescence colocalization experiments. This reduction in colocalization is consistent with decreased signal transduction from upstream to downstream components within the pathway, thereby inhibiting the activity of the PI3K–AKT signaling pathway. We acknowledge that immunofluorescence colocalization does not demonstrate direct PPI; future studies using co‐immunoprecipitation or PLA are needed to further investigate the mechanism. Concurrently, rescue experiments revealed that co‐administration of triptolide and the PI3K activator 740Y‐P effectively reversed the phenotypic alterations induced in TSCC cells. While our rescue experiments using the PI3K agonist 740Y‐P support pathway specificity, future studies employing constitutively active AKT (e.g., Myr‐AKT), rather than wild‐type AKT, could provide further genetic validation by bypassing upstream PI3K‐dependent phosphorylation. From molecular binding and PPIs to functional phenotypes, this study rigorously demonstrates at multiple levels that triptolide exerts a key mediating role in inhibiting the proliferation, invasion, migration, and EMT of TSCC cells by regulating the potential PI3K–AKT pathway.

Admittedly, the anti‐tumor mechanism of triptolide is complex and multifaceted. Our research has for the first time systematically elucidated and experimentally validated the pivotal role of the PI3K–AKT pathway in the inhibition of TSCC by this compound. This provides a novel theoretical foundation for developing targeted therapeutic strategies against this specific cancer. The present work is an essential but preliminary step. Future studies must expand the mechanistic canvas—addressing, for example, feedback loops involving mTORC2, SGK, or ILK, and examining immune‐modulatory consequences within the tumor microenvironment. Clinical translation will also require rigorous optimisation of formulation and dosing. Minnelide, lipidosomes, and hyaluronic‐acid‐decorated nanoparticles already offer proven strategies to circumvent solubility and toxicity barriers; incorporation of triptolide into such carrier systems, or its coformulation with subtherapeutic cisplatin, may permit efficacious exposure while sparing normal mucosa. In‐depth pharmacokinetic profiling and long‐term safety evaluation in relevant large‐animal models are therefore the logical next milestones on the path toward investigator‐initiated trials in TSCC patients.

## Funding

National Natural Science Foundation of China (NSFC82360958, NSFC82560962). This project partially supported the additional experiments performed for this study. Natural Science Foundation of Jiangxi Province (20224ACB206039); General Project of Science and Technology Plan of Jiangxi Administration of Traditional Chinese Medicine (2023B1211).

## Conflicts of Interest

The authors declare no conflicts of interest.

## Supporting information


**Figures S1–S3:** Effects of triptolide on TSCC cell invasion (S1), colony formation (S2), and migration (S3) at sublethal concentration (1/4 IC_50_).

**Figure S4:** Pharmacological validation of PI3K–AKT pathway involvement.

**Table S1:** Binding kinetics of triptolide with AKT1 and PI3K determined by surface plasmon resonance (SPR).

## Data Availability

The data that support the findings of this study are available from the corresponding author upon reasonable request.
